# Nutrient intake, digestibility, performance, carcass traits and sensory analysis of meat from lambs fed with co-products of Amazon oilseeds

**DOI:** 10.3389/fvets.2023.1181765

**Published:** 2023-05-25

**Authors:** Vinicius Costa Gomes de Castro, Juliana Cristina de Castro Budel, Thomaz Cyro Guimarães de Carvalho Rodrigues, Bruna Almeida Silva, Alyne Cristina Sodré de Lima, Shirley Motta de Souza, Jamile Andréa Rodrigues da Silva, Maria Regina Sarkis Peixoto Joele, André Guimarães Maciel e Silva, José de Brito Lourenço-Junior

**Affiliations:** ^1^Postgraduate Program in Animal Health and Production in the Amazon, Federal Rural University of the Amazon, Pará, Brazil; ^2^Institute of Veterinary Medicine, Federal University of Pará, Pará, Brazil; ^3^Department of Food Technology, State University of Pará, Pará, Brazil; ^4^Federal Institute of Amapá, Amapá, Brazil; ^5^Department of Animal Science, Federal Institute of the South of Minas Gerais, Minas Gerais, Brazil; ^6^Federal Institute of Pará, Pará, Brazil

**Keywords:** agroindustry byproducts, ruminant nutrition, cupuassu, palm kernel, sheep, sustainability, tucuma

## Abstract

**Introduction:**

The increase in availability and nutritional composition of oilseed co-products has made it essential to study the use of this biomass.

**Methods:**

The objective of this work was to investigate the effects of including oilseed cakes on intake and digestibility, performance, carcass characteristics and meat sensory in feedlot lambs. Twenty-four crossbred Dorper × Santa Inês lambs, with initial body weight of 30 ± 1.3 kg, male, castrated, aged 4–5 months, were distributed in a completely randomized experimental design with four treatments (diets) and six replications (animals), confined in individual stalls for 70 days.

**Results:**

The inclusion of tucuma cake (Tuc) reduced dry matter intake (*p* < 0.01) and diets with cupuassu cake (Cup) and palm kernel cake (Palm) reduced dry matter digestibility (*p* < 0.05). The Tuc diet also provided the lowest final body weight (*p* = 0.02); lower average daily gain (*p* = 0.03); lower feed efficiency (*p* = 0.03) and lower carcass weight (*p* < 0.01). However, diets did not influence carcass yield (%), fat thickness (mm) and loin eye area (cm^2^; *p* > 0.05). Meat from lambs on the control diet was rated as less fibrous and more tender (*p* < 0.05).

**Conclusion:**

The inclusion of tucuma cake does not influence digestibility, but reduces intake, performance and influences carcass characteristics and meat texture. Diets with cupuassu cake or palmiste cake reduced digestibility, however, intake, performance and carcass characteristics were similar to the control diet.

## 1. Introduction

The reduction of industrial waste is a worldwide trend that has led to studies on the use of this material in animal feed (1). The recovery and valorization of this biomass through the use in diets for ruminants, drives the advance of the agroindustry towards models of circular economy and sustainable production (2). In addition, it allows the replacement of edible food crops by inedible biomass and the reduction in production costs (3).

Oilseed cakes are an example of this previously discarded biomass. They are generated from the oil extraction process, to meet the growing demand of the sectors: pharmaceutical; to feed; biofuels and cosmetics (4). The inclusion of these co-products in ruminant feed has been studied due to the levels of neutral detergent fiber, crude protein and energy, through the residual ether extract, and show promising results (5–8), including in combined inclusion (9).

In the Amazon region, we can highlight the production of cupuassu (*Theobroma grandiflorum Schum*), palm kernel (*Elaeis guineenses*) and tucuma (*Astrocaryum aculeatum*), which generate a high volume of cake, but which present 7.4–16.2% of EE; 8.5–18.7% CP and 53.1–69% NDF and potential for inclusion in diets for ruminants (10–12). The inclusion of cupuassu did not influence the consumption, production and composition of buffalo milk (6); the addition of 12% palm kernel cake increased intake, performance, marbling and feed efficiency in kids (13) and did not change the qualitative carcass attributes in Nellore cattle (14); the inclusion of 45% tucuma cake provided results similar to control treatments in confined sheep (12).

The sheep herd in Brazil has increased by 10% in the last 5 years, exceeding 20 million animals, but still insufficient to meet the national demand (15). The use of co-products as an ingredient in their diet, in addition to improving the energy efficiency of the diet and reducing the inappropriate disposal of biomass, is a strategy that can contribute to the productivity of the chain. However, the effects of cupuassu, palm kernel and tucuma cakes on intake, digestibility, performance, carcass traits and meat acceptance in lambs need to be investigated.

Our hypothesis is that the inclusion of oilseed cakes does not compromise intake, digestibility and performance, but improves carcass traits and meat acceptance. Thus, the aim of this study was to investigate the effects of including oilseed cakes on these parameters in feedlot lambs.

## 2. Materials and methods

The research was approved by the Ethics Committee for the Use of Animals (CEUA) of the Federal University of Pará—protocol 8,694,141,217.

### 2.1. Location, animals and diets

The experiment was conducted in the experimental installation of the Federal Institute of Education, Science and Technology of Pará—IFPA (1°18′10.08′′ S, 47°56′56.10′′ W), Castanhal, Pará, with climate type Af, according to Köppen, and average precipitation of 2.770 mm/year, average annual temperature and relative humidity air 26.8°C and 85%, respectively.

Twenty-four crossbred Dorper × Santa Inês lambs, with initial body weight of 30 ± 1.30 kg, male, castrated, aged between 4 and 5 months, were distributed in a completely randomized experimental design with four treatments (diets) and six replications (animals). The animals were housed in individual pens measuring 1.5 m^2^ (1.0 × 1.5 m), covered, with a wooden slatted floor and suspended, provided with drinkers and feeders with unrestricted access to water and experimental diets. The stalls were in a covered shed, with a side opening for natural wind circulation. At the beginning of the experiment, the animals were identified and subjected to parasite control.

The experimental period lasted 70 days, preceded by 10 days for the animals to adapt to the environment, stalls, management and diets. The control diet (Control) contained ground corn and soybean meal as concentrate ingredients, which were partially replaced by cupuassu cake (Cup diet), palm kernel cake (Palm diet) and tucuma cake (Tuc diet). The three diets with co-products from the Amazon agroindustry were balanced to be isoproteic and isoenergetic, in order to evaluate the potency of the co-products in substitution of the control treatment. The four diets included 400 g/kg of dry matter (DM) of corn silage as forage component and 600 g/kg DM of concentrated component differing in ingredients (inclusion of co-products), homogenized and offered as total diet (Table 1).

**Table 1:** Ingredients and chemical composition of the experimental diets.

Items	Diets			
	Control	Cup	Palm	Tuc
*Ingredients (g/kg DM)*
Corn silage	400	400	400	400
Ground corn	432	62	260	132
Soybean meal	148	68	144	139
Cupuassu seed cake	-	450	-	-
Palm kernel cake	-	-	176	-
Tucuma kernel cake	-	-	-	309
Mineral-vitamin premix^1^	15	15	15	15
Limestone	5	5	5	5
*Chemical composition (g/kg DM)*
Dry matter (g/kg as fed)	686	687	690	687
Ash	47.9	55.1	51.0	47.1
Crude protein	142	144	142	146
Ether extract	63.8	66.2	66.9	69.9
NDFcp^2^	308	384	385	445
ADFcp^3^	138	193	215	256
Lignin	132	107	100	77
Total digestible nutrients	691	680	679	679

Diets: Cup, cupuassu kernel cake; Palm, Palm kernel cake; Tuc, tucuma kernel cake. ^1^Calcium 140 g. Phosphorus 65 g. Magnesium 10 g. Sulfur 12 g. Sodium 130 g. Cobalt 80 mg. Iron 1.000 mg. Iodine 60 mg. Manganese 3.000 mg. Selenium 10 mg. Zinc 5.000 mg. Fluor (max) 650 mg. Vitamin A 50.000 IU. Vitamin E 312 IU. ^2^Neutral detergent fiber corrected for ash and protein. ^3^Acid detergent fiber corrected for ash and protein.

The diets were formulated according to the recommendations of the National Research Council (16) in order to meet the requirements for lambs and allow an estimated average daily weight gain of 200 g. They were provided in two meals, 50% at 7:30 am and the other 50% at 4:30 pm.

### 2.2. Intake, apparent digestibility of nutrients and chemical analysis

Consumption of nutritional components was determined by the difference between the amount of each component contained in the feed provided and the amount contained in leftovers. Daily, before offering the morning diet, leftovers were collected and weighed to determine dry matter intake and feed adjustment to allow 10–15% leftovers. In addition, samples of diet ingredients and leftovers were collected once a week. Subsequently, these samples were proportionally grouped to obtain the composite sample and placed in identified plastic bags.

Diet digestibility was estimated using indigestible NDF as an internal marker (17). For this purpose, feces were collected directly from the rectal ampulla during five consecutive days and at different times (0, 3, 6, and 9 h after the first feed offer), aiming at greater daily representativeness. They were then identified and stored in a freezer at −8°C. At the end of data collection, the material obtained in the 5 days of collection was homogenized, forming a composite sample. Then, they were dried in a forced ventilation oven (55°C) until constant weight. The digestibility coefficient was estimated using an internal indicator: indigestible insoluble neutral detergent fiber (NDi). The offered diets, leftovers and faeces were incubated in situ, using TNT bags, in the proportion of 20 mg of DM cm^2^, in triplicate, in the rumen of crossbred Murrah-Mediterranean buffaloes, for 240 h (18). After the incubation period, analysis of the insoluble fiber in neutral detergent was performed to quantify the NDFi levels. Fecal excretion values were calculated by the relationship between consumption and fecal concentration of NDFi.

Feeds, ingredients and leftovers were collected and they were pre-dried in an oven with forced air circulation at 55°C for 72 h. Then, the samples were ground in a Willey-type mill with a one-mm sieve sieve to determine for dry matter (MS—method G-003/1), ash (method M-001/1), crude protein (CP—method N-001/1), ether extract (EE—method G-005/1), neutral detergent fiber corrected for ash and protein (NDFap—method F-002/1) and acid detergent fiber corrected for ash and protein (ADFap—F-004/1), lignin (method F-005/01) using the methods recommended by the National Institute of Science and Technology in Zootechnics—INCT-CA (17).

Digestibility coefficients (DC) were calculated using the following equation:

DC = [(Amount ingested—Amount excreted)/(Amount ingested)] × 100.

### 2.3. Performance

The productive performance of the lambs was evaluated through the individual weighing of the animals on the first experimental day and on the last day, always in the morning, before the supply of the first meal.

Thus, total weight gain (TWG) was determined by the difference in final and initial body weight; and the average daily gain (ADG) by dividing the TWG by the number of confinement days (TWG/70). With the total daily intake of dry matter (total DMI) and the average daily gain (ADG), the feed efficiency (FE) was calculated using the following formula: FE = ADG/total DMI. Where: total DMI = total daily intake of dry matter; TWG = daily weight gain, in kg/day; and FE = feed efficiency.

### 2.4. Slaughter and carcass characteristics

On the 71st day of confinement, the animals were sent to the experimental slaughterhouse of the Instituto Federal do Pará (IFPA), Castanhal, Pará, Brazil. They underwent a period of fasting and rest at 4 pm and then weighed to obtain slaughter body weight (BW).

At the time of slaughter, the animals were stunned by electronarcosis, followed by bleeding, skinning and evisceration, respecting the procedures for handling and humane slaughter of animals (19). At the end of the slaughter, the carcasses were weighed obtaining the weight and yield of the hot carcass (HCW and HCY = HCW/CWS*100), then taken to the cold room at a temperature of 6°C, where they remained for 24 h hung by the tendons of the Gastrocnemius muscle on appropriate hooks. After this period, they were weighed again to obtain cold carcass weight and yield (CCW and CCY = (CCW/CWS)*100).

Subsequently, in the loins (*Longissimus lumborum*), the loin-eye area (LEA) was determined from a cross-section between the 12th and 13th thoracic vertebrae, which was performed using sheets of plastic transparency for the design of the area, in correspondence with the cranial portion of the loin. Thus, the following measurements were established: The length (A) and maximum depth (B) of the *L. lumborum* muscle, in cm, measured with the aid of a ruler and calculated from the ellipse formula: LEA = (A/2* B/2) π, in cm^2^, proposed by Silva Sobrinho (20). The subcutaneous fat thickness (SFT) in the carcass was measured, in mm, with the aid of a digital caliper at ¾ of a distance from the medial side of the *L. lumborum* muscle, to the side of the spinous apophysis.

Subsequently, the loins (right and left) of each animal were packaged, identified and frozen in a freezer (−20°C) for further analysis of sensory evaluation.

### 2.5. Sensory evaluation

Before sensory analysis, the microbiological quality of the meat was assessed and considered safe for sensory testing.

The evaluators were recruited after applying a questionnaire consisting of questions about availability to participate in the research, eating habits, including dietary restrictions associated with health, religious beliefs, diets and medication use. Fifty possible evaluators were recruited, including students from the Food Technology class at the State University of Pará and employees of the Federal Institute of Education, Science and Technology of Pará, who participated in aroma, flavor and texture recognition tests to verify the ability to recognize attributes (21). Of the 50 recruited, 20 tasters were selected who answered more than 70% of the questionnaire with greater discriminatory power and reproducibility of results. The group final of evaluators was composed of 10 women and 10 men aged between 18 and 38 years.

The meat was wrapped in aluminum foil and baked in an oven until it reached an internal temperature of 70°C, measured with a thermocouple. The samples were cut parallel to the muscle fibers (1.5 cm cubes), wrapped in aluminum foil, coded with 3 digits and served at 60°C (22). Sensory analysis was performed individually and under controlled environmental conditions.

The attributes were determined according to the Quantitative Descriptive Analysis (QDA) and the description of the terminology was carried out after the tasters’ assessment (ISO 8586:2012). Each evaluator received the meat samples (Control, Cup, Palma, and Tuc) and a card with an unstructured hedonic scale, where 0 refers to the minimum intensity and 9 to the maximum for each attribute. Each evaluator indicated with a vertical line below the scale line the point that best represented the perceived intensity (appearance, aroma, flavor and texture) in the meat (23). The evaluator was instructed to drink mineral water between tastings to clean the taste buds and minimize residual effects.

### 2.6. Statistical analysis

The variables under study were submitted to the Shapiro–Wilks normality test, and in case they did not follow a normal distribution, the variables were analyzed using non-parametric statistics (Kruskal–Wallis, Friedman). Subsequently, analysis of variance was performed considering the completely randomized design with a fixed factor (diet—four levels), for the variables following normal distribution.

The effect of diets on intake, digestibility, performance and carcass variables including initial weight as a covariate (*p* < 0.05), according to the statistical model: Yij = *μ* + *C* + NSi + eij. Where: Yij = observed value of characteristic *Y*, *i* repetition and *j* diets; *μ* = overall mean; *C* = initial weight covariate (kg); NSi = treatment-related effect; eij = random error, associated with each Yij observation. Least squares adjusted means (24) were compared using Tukey’s test (*p* < 0.05).

The data referring to the sensory analysis were submitted to statistical analysis considering the levels of inclusion of the Amazon cakes as a fixed effect and evaluators as a random effect. Thus, the Poisson distribution was used through PROC GLIMMIX of SAS 9.1, considering in all evaluations up to 5% probability for type I error.

## 3. Results

### 3.1. Nutrient intake and apparent digestibility

Dry matter intake (g/day) was higher in Control (1,193) and Cup (1,261) treatments, followed by Palm (1,071) and Tuc (858; *p* < 0.01). Crude protein intake (g/day) was higher in the control treatment (255), the same for animals on the Cup (218) and Pal (189) diets, and lower for Tuc (172; *p* = 0.03). Sheep on the Tuc diet also had a lower intake of ether extract (45 g/day), control and Palm did not differ from each other (79 and 76 g/day) and Cup provided a higher intake (99 g/kg; *p* < 0.01). NDF consumption did not differ between treatments, averaging 446.75 g/day (*p* > 0.05) (Table 2).

**Table 2:** Nutrient intake and apparent digestibility of lambs fed by-products of Amazonian oilseeds.

Item	Diet	SEM	*p*-Value
	Control			Cup	Palm	Tuc
Intake (g/day)
DM	1193^a^	1261^a^	1071^ab^	858^b^	0.06	<0.01
CP	255^a^	218^ab^	189^ab^	172^b^	0.01	0.03
EE	76^b^	99^a^	79^b^	45^c^	0.01	<0.01
NDF	379	469	457	482	0.02	0.11
Digestibility (g/kg)
DM	838^a^	761^b^	698^b^	844^a^	1.62	<0.01
CP	860^a^	760^b^	815^ab^	828^a^	1.58	<0.01
EE	879	887	915	901	0.83	0.78
NDF	751^a^	664^b^	657^b^	749^a^	2.12	<0.01

Cup, diet with cupuassu cake; Palm, diet with palm kernel cake; Tuc, diet with tucuma cake; SEM, standard error of the mean; DM, dry matter; CP, crude protein; EE, ether extract and NDF, neutral detergent fiber.

The DM digestibility (g/kg) was higher in Control (838) and Tuc (844) treatments, followed by Cup (761) and Palm (698; *p* < 0.01). The CP digestibility (g/kg) was also higher in Control (860) and Tuc (828), followed by Palm (815) and Cup (760; *p* < 0.01). There was no effect of the diets on the EE digestibility, however, for NDF, the Control (751) and Tuc (749) treatments also obtained the highest values, followed by Cup (664) and Palm (657 g/day; *p* < 0.01).

### 3.2. Performance

The variation of 2.1 kg in the initial weight of the lambs demonstrates the homogeneity of the confined animals for the research (Table 3). The final weight (kg) of lambs fed the control diet was higher (38.8), Cup and Palm did not differ from each other (37.3 and 35.9, respectively), and Tuc had the lowest value (34; *p* = 0.02). For the average daily gain, the behavior was the same, with the highest value (g/day) obtained by the control diet (124), followed by Cup (102) and Palm (93) which did not differ, and the lowest value obtained by the lambs Tuc (62; *p* < 0.03). The diet that provided the highest feed efficiency was the control (0.10), followed by the Cup (0.08) and Palm (0.08) treatments, and the lowest efficiency was observed in animals fed with Tuc (0.06; *p* = 0.03).

**Table 3:** Performance of lambs fed by-products of Amazon oilseeds.

Item	Diet	SEM	*P*-value
	Control			Cup	Palm	Tuc
Initial BW (kg)	29.3	30.4	31.3	29.1	-	-
Final BW (kg)	38.8^a^	37.3^ab^	35.9^ab^	34.0^b^	0.81	0.02
ADG (g/day)	124^a^	102^ab^	93^ab^	62^b^	0.01	0.03
FE	0,10^a^	0,08^ab^	0,08^ab^	0,6^b^	0.31	0.03

Cup, diet with cupuassu cake bran; Palm, diet with palm kernel cake bran; Tuc, diet with tucuma cake bran; SEM, standard error of the mean; BW, body weight; ADG, average daily gain; FE, feed efficiency.

### 3.3. Carcass characteristics

Only the Tuc diet differed in hot carcass weight (kg), where the lowest value (16.0) was observed, in relation to the other treatments (control 19.1; Cup 18.8; Palm 18.0; *p* < 0.01) (Table 4). The same behavior was observed in the evaluation of cold carcass weight, with lower weight (kg) for animals fed Tuc (15.6), followed by control (18.8), Cup (18.8) and Palm (17.7), which did not differ (*p* < 0.01). There was no effect of diets on carcass yield (%), fat thickness (mm) and ribeye area (cm^2^; *p* > 0.05).

**Table 4:** Carcass characteristics of lambs fed by-products of Amazon oilseeds.

Item	Diet	SEM^1^	*P*-value
	Control			Cup	Palm	Tuc
Hot carcass (kg)	19.1^a^	18.8^a^	18.0^a^	16.0^b^	0.47	<0.01
Cold carcass (kg)	18.8^a^	18.5^a^	17.7^a^	15.6^b^	0.47	<0.01
Carcass yield (%)	50.1	50.1	48.1	48.3	0.51	0.57
SFT (mm)	1.47	1.4	1.43	1.41	0.02	0.18
LEA (cm^2^)	13	13.9	13.1	11.8	0.37	0.88

Cup, diet with cupuassu cake bran; Palm, diet with palm kernel cake bran; Tuc, diet with tucuma cake bran; SEM. standard error of the mean; SFT, subcutaneous fat thickness; LEA, loin eye area.

### 3.4. Sensory evaluation

There was no influence of treatments on appearance (color and presence of nerves); aroma (meat, blood and fat) and flavor (meat, blood and fat; *p* > 0.05). However, for texture data, tenderness was higher in the control diet (6.12), followed by Cup (5.04), Tuc (3.78) and Palm (3.06; *p* = 0.02). Meat judged as more fibrous was the Tuc diet (6.03), followed by Palm (5.13), Cup (3.6) and control (3.24; *p* = 0.02). Juiciness was not affected by diets (*p* > 0.05).

## 4. Discussion

### 4.1. Intake and digestibility

The lower DMI observed in sheep fed the Tuc diet was possibly due to three factors: the EE content of the diet (6.99%); the highest level of NDF (44.5; Table 1); the reduced acceptance of the animals to the new ingredient. The inclusion of the co-product totaled 30.9% of the total diet, a significant amount that can be reassessed in future studies (Table 2).

The negative effects of oilseed co-products in ruminant diets may be related to increased levels of fat and fiber (3). In ruminants, the variation of only one of the sensory characteristics of the diet is enough to change the feed intake of the animals (25). Systematic studies investigate the taste preference of ruminants, aiming to provide information on the acceptance of synthetic blends and flavors (26).

With a reduction in DM intake, the Tuc animals also had lower CP and EE intake. The intake of DM influences the intake of nutrients, since it is composed of the sum of these. Nutrient intake and animal performance are directly linked to dry matter intake (27).

Despite the distance of up to 13.7% in fiber content between the diets, there was no difference in NDF consumption. This was probably due to the selection of animals, preferring the portion with greater or lesser fiber content. Small ruminants are selective animals regarding diets and make their choices according to availability and acceptability (28). In a study evaluating the inclusion of licuri cake in diets for kids, (29) observed that NDF consumption was higher in the control diet, compared to the diet with a higher level of the co-product, which had a higher fiber.

The control and Tuc diets obtained the highest DM digestibility coefficients and this was possibly due to the higher TDN content of the control diet and, in the case of Tuc, a lower percentage of lignin and a higher percentage of EE (Table 1). In addition, the CP of these diets (control and Tuc) was also more digestible. The EE digestibility was not influenced by the diets, allowing us to state that the residual fat present in the co-products of the oilseeds is of equal quality to that of the conventional ingredients (ground corn and soybean meal). The highest percentage of EE in the Tuc diet also did not influence the NDF digestibility, where it obtained the highest result with the control diet.

Variations in the total digestible content and metabolism of nutrients result in different availability in the body of animals (30). The inclusion of co-products, due to their fibrous content, has a great influence on the digestibility of diets (31–33).

### 4.2. Performance

The lowest final weight and ADG was observed on the Tuc diet, which, despite also having the lowest initial weight, promoted the lowest average daily gain (Table 3). This fact occurred due to the lower consumption observed for this treatment (Tables 1, 2). Even with digestibility values similar to the control treatment, the consumption of Tuc animals was 28.2% lower. Intake, when diets are similar, is the best indicator of weight gain (34). When the feed is of good quality and optimizes intake levels, animal performance can be substantially increased (35).

Animals with low consumption, but with the same level of production as the others, have differentiated genetics (naturally more efficient), or received a best quality diet, where the use was more effective (36). In the present work, as there was no genetic differentiation and changes in diets with the inclusion of oilseed cakes influenced consumption and digestibility, there was a difference in the feed efficiency of the animals.

### 4.3. Carcass characteristics

The difference in final weight between treatments had a direct influence on carcass weight, with animals on the Tuc diet having the lowest values for hot and cold carcass (Table 4). However, the differences observed for weight gain and carcass weights did not influence the carcass yield, suggesting that the composition of the gain was proportional regardless of the diet, since the fat thickness and rib eye area were also the same (37).

The deposition of Protein and fat depend on factors such as: age, sexual class, hormonal regulation (endogenous and exogenous) and intake of necessary nutrients, mainly through the effects of the total energy absorbed and available for daily needs and growth (38). Consumption of formulated diets improves the prediction of weight gain and body composition compared to other feeding systems (39).

Measurement of LEA has been performed (*in vivo*) by ultrasonography, as it is efficient in monitoring marketable meat gain, animal performance and genetic selection (40). Higher LEA values are indicative of greater efficiency and feed conversion into product and performance, and result in heavier carcasses (41, 42).

### 4.4. Sensory evaluation

Feed management practices influence specific nutritional, technological and sensory quality characteristics of ruminant products (43). The evaluation of this influence on sensory attributes and consumer acceptance of possible changes such as flavor tenderness and juiciness, are essential (44, 45).

However, appearance, aroma and flavor were attributes that were not influenced by the inclusion of cakes (Table 5). The influence was on the fibrosity and texture of the meat, which resulted in greater tenderness and less fibrousness in the animals on the control diet. The lack of effect on juiciness reinforces the equality in fat thickness data, directly associated with meat juiciness (46, 47). Tenderness is an important meat quality trait and determines satisfaction, repeat purchase and willingness to pay premium prices, however diet is just one of many factors that can influence this characteristic (48, 49). The scores for the sensory attributes in this study were higher than those observed by Silva et al. (50); and lower than those found by Ribeiro et al. (51) and Silva et al. (6) in meat from confined goats fed diets containing cakes of peanult, palmiste, and licuri, respectively.

**Table 5:** Sensory attributes of meat from lambs fed with by-products of Amazon oilseeds.

Item	Diet				*P*-value
	Control	Cup	Palm	Tuc	
*Appearance*
Color	5.13	4.86	4.59	3.42	0.36
Presence of nerve	4.14	5.4	4.5	3.96	0.51
*Aroma*
Lamb meat	4.05	5.76	3.42	4.77	0.12
Blood	3.96	4.68	5.85	3.51	0.10
Fat	4.68	4.5	4.23	4.59	0.97
*Flavor*
Lamb meat	4.14	4.59	4.41	4.86	0.92
Blood	5.4	3.87	4.41	4.32	0.50
Fat	3.79	5.13	3.97	5.31	0.25
*Texture*
Tenderness	6.12^a^	5.04^ab^	3.06^c^	3.78^bc^	0.02
Fibrosity	3.24^c^	3.6^bc^	5.13^ab^	6.03^a^	0.02
Juiciness	3.78	3.6	6.03	4.59	0.07

Qualitative values refer to a hedonic scale from 0 to 9. Cup, diet with cupuassu cake bran; Palm, diet with palm kernel cake bran; Tuc, diet with tucuma cake bran; SEM, standard error of the mean.

## 5. Conclusion

The inclusion of tucuma cake does not influence digestibility, but reduces intake, performance and influences carcass characteristics and meat texture. Diets with cake of cupuassu and palmiste reduced digestibility, however, intake, performance and carcass characteristics were similar to the control diet. The use of CUP and PALM is indicated for medium performances, up to 120 g/day and TUC is not indicated as the main ingredient in finishing lamb diets because it limits the consumption and performance of the animals. We suggest that further research be carried out to verify the results and improve the use of this cakes in lambs feed.

## Data availability statement

The original contributions presented in the study are included in the article/supplementary material, further inquiries can be directed to the corresponding author.

## Ethics statement

The animal study was reviewed and approved by Ethics Committee for the Use of Animals (CEUA) of the Federal University of Pará.

## Author contributions

AS, JL-J, and JS: experiment design. VC, JB, and BS: experiment perform. JS, TR, AS, and JL-J: data curation. AL, MJ, AS, and SS: formal analysis. VC, TR, AS, and JL-J: writing-original draft. All authors contributed to the article and approved the submitted version.

## Funding

This work was supported by Coordination for the Improvement of Higher Education Personnel (CAPES) and Dean of Research and Graduate Studies (PROPESP/UFPA).

## Conflict of interest

The authors declare that the research was conducted in the absence of any commercial or financial relationships that could be construed as a potential conflict of interest.

## Publisher’s note

All claims expressed in this article are solely those of the authors and do not necessarily represent those of their affiliated organizations, or those of the publisher, the editors and the reviewers. Any product that may be evaluated in this article, or claim that may be made by its manufacturer, is not guaranteed or endorsed by the publisher.
